# Exploiting the heightened phase synchrony in patients with neuromuscular disease for the establishment of efficient motor imagery BCIs

**DOI:** 10.1186/s12984-018-0431-6

**Published:** 2018-10-29

**Authors:** Kostas Georgiadis, Nikos Laskaris, Spiros Nikolopoulos, Ioannis Kompatsiaris

**Affiliations:** 1 AIIA lab, Informatics Department, AUTH, Thessaloniki, Greece; 2 Information Technologies Institute (ITI), Centre for Research & Technology Hellas, Thessaloniki-Thermi, Greece; 3 NeuroInformatics.GRoup, AUTH, Thessaloniki, Greece

**Keywords:** Phase synchrony, Chronnectomic patterns, BCI, Classifier-ensembles

## Abstract

**Background:**

Phase synchrony has extensively been studied for understanding neural coordination in health and disease. There are a few studies concerning the implications in the context of BCIs, but its potential for establishing a communication channel in patients suffering from neuromuscular disorders remains totally unexplored. We investigate, here, this possibility by estimating the time-resolved phase connectivity patterns induced during a motor imagery (MI) task and adopting a supervised learning scheme to recover the subject’s intention from the streaming data.

**Methods:**

Electroencephalographic activity from six patients suffering from neuromuscular disease (NMD) and six healthy individuals was recorded during two randomly alternating, externally cued, MI tasks (clenching either left or right fist) and a rest condition. The metric of Phase locking value (PLV) was used to describe the functional coupling between all recording sites. The functional connectivity patterns and the associate network organization was first compared between the two cohorts. Next, working at the level of individual patients, we trained support vector machines (SVMs) to discriminate between “left” and “right” based on different instantiations of connectivity patterns (depending on the encountered brain rhythm and the temporal interval). Finally, we designed and realized a novel brain decoding scheme that could interpret the intention from streaming connectivity patterns, based on an ensemble of SVMs.

**Results:**

The group-level analysis revealed increased phase synchrony and richer network organization in patients. This trend was also seen in the performance of the employed classifiers. Time-resolved connectivity led to superior performance, with distinct SVMs acting as local experts, specialized in the patterning emerged within specific temporal windows (defined with respect to the external trigger). This empirical finding was further exploited in implementing a decoding scheme that can be activated without the need of the precise timing of a trigger.

**Conclusion:**

The increased phase synchrony in NMD patients can turn to a valuable tool for MI decoding. Considering the fast implementation for the PLV pattern computation in multichannel signals, we can envision the development of efficient personalized BCI systems in assistance of these patients.

**Electronic supplementary material:**

The online version of this article (10.1186/s12984-018-0431-6) contains supplementary material, which is available to authorized users.

## Background

According to World Health Organization (WHO) approximately 15% of the global population experiences some kind of disability with a 2–4% being reported as severe.^1^ Brain Computer Interfaces (BCIs) receive continuous attention as an emerging technology for rehabilitation and restoration of communication in people with disabilities. BCIs create a communication channel between the brain and machines, such as computers, as they “translate” brain signals into machine commands without requiring any muscle or peripheral nerve activity [1, 2]. The idea of “mind reading” was first conceived by Berger [3], but only in the past few years BCI implementations were made plausible. BCIs can be implemented with various approaches, but electroencephalography (EEG) has been proven to be the most popular choice due to its non-invasiveness, low cost and advantage of being employed with minimal effort even in home environments.

EEG-based BCIs can be categorized as exogenous or endogenous depending on whether external stimulation is provided to the user. Event-related (evoked) potential, ERP(EP), BCIs belong to the exogenous BCIs as the brain activation is measured after a specific event (or delivered stimulus). Most often visual stimuli are encountered, since they are more naturally perceived, with the most notable examples being transient [4], code-modulated [5] and steady-state [6, 7] visual responses to flickering patterns. While exogenous BCIs achieve high performance, their design inherently contradicts with the perspective of asynchronous (i.e. self-paced) BCIs, and this is the main reason why endogenous BCIs currently receive significant attention, even though a considerable training period, that can last from a couple of days to several months, is required for the user before harnessing such a system. The most prominent paradigm of endogenous BCIs is the one that requires the user to perform a mental task, including movement imagination of limb(s) or even tongue [8–12], speech imagination [13, 14] and mental arithmetic [15, 16]. In the case of movement imagination, called hereafter motor imagery (MI), particularly, brain decoding usually relies on the sensorimotor rhythm (SMR) detected in the EEG signal from the electrodes located over the sensory-motor cortex, the part of the brain that is associated with planning, control and execution of voluntary movements [17].

MI related modulations in brain activity, associated with both μ and β rhythms over the sensorimotor areas are often reported in EEG studies and the approach of event-related desynchronization/synchronization (ERD/ERS) that estimates the power increase/decrease during the MI task or once it is completed, has been developed to capture them [18–20]. A second popular approach is the technique of common spatial patterns (CSPs) [21] and its alternatives [22–25], where spatial filtering is combined with classification so as to decode the intended movement. Signal-amplitude characteristics, derived in the time domain, are exploited in all these approaches. Phase synchrony has recently entered into the picture and led to novel alternative ways in decoding an indented movement by describing the functional inter-areal interactions during MI [26, 27]. The metric of phase locking value (PLV) is usually employed and features from either the static or dynamic connectivity patterns, as they emerge over the sensor space, have been demonstrated to facilitate the effective decoding of user’s intentions [28, 29].

In the related literature of MI-BCIs, there are only a few studies that deal with the option of a self-initiated motion. In two of them, Scherer et al. [30] and Chae et al. [31], a two-stage classification scheme is adopted. The first stage takes over the detection of (the onset of) an MI-event, while the second stage performs the final read out (i.e. the direction of the movement). Additionally, a “brain switch” has been implemented based on the β rhythm rebound (i.e. ERS) that appears at the end of a particular MI event. Either a simple thresholding scheme [32] or linear discriminant analysis [33] is employed to flag a significant departure from the ongoing activity that corresponds to an “idling” (baseline) state. Once an MI event is detected, the associated command is given to the actuator.

The above mentioned MI-BCI approaches have been investigated in several studies with participants suffering from motor disabilities, including amyotrophic lateral sclerosis (ALS) [34, 35], spinal cord injury (SCI) [36, 37], multiple sclerosis (MS) [38, 39] and chronic strokes (CS) [40, 41]. However, only a limited number of studies have been done on people suffering from neuromuscular disease (NMD) [42]. In contrast with SCI and CS, NMD is a progressive condition that often initiates with the affection of specific group of muscles and finally spreads to many other groups, resulting in gradual loss of a patient’s fine motor skills. Therefore, significant mental effort is required by the patients to make a move or even attempt to move their limbs in their everyday life for several years, prior to the complete loss of their movement control. In this direction, the initial motivation of this study was to examine how NMD-patients, as novice BCI users, would perform in simple MI tasks (imagination of left/right hand movement) without any training and/or feedback. We hypothesized that, due to long-lasting self-organization, phase synchrony would govern their re-configured brain networks and could be detected in the sensor space when they were cued to imagine a limb movement (which for them is almost equivalent to try to realize the same movement).

The contribution of this paper is threefold. We first show that NMD patients are characterized by increased level of both phase-synchrony and network organization with respect to healthy controls. Next, we demonstrate a nearly optimal performance for a decoding scheme that exploits, in a personalized fashion, the connectivity patterns emerging during cued imaginary movement. Finally, we describe a novel algorithmic procedure that could adapt the proposed decoding scheme for a self-paced BCI scenario and provide a “proof-of-concept” using the available data. Besides the documented effectiveness, our proposal is supported by the computational efficiency of the adopted PLV implementation (see Appendix).

## Methods

### Participants

A total of twelve individuals (7 males and 5 females, aged 36.08 ± 6.45) participated in this study, separated into two groups. More specifically, the first group consists of six people suffering from NMD and the second of six able-bodied with a matching socio-demographic profile. Table 1 provides information about each participant, while a more detailed description (e.g. inclusion criteria, clinical characteristics) can be found in [43]. All subjects had normal or corrected-to-normal vision and none of them had taken any psycho-active or psycho-tropic substance. Participants had no prior experience with SMR protocols, or any other BCI protocol. Prior to the experimental session, subjects and their caretakers were informed about the experimental procedure. A consent form, thoroughly read, was signed by the participants or in cases of inability by their caretakers. The experimental protocol was approved by the Ethical Committee of MDA HELLAS.

**Table 1:** Subject Demographics

Able-bodied subjects			NMD patients			
Participant ID	Gender	Age	Participant ID	Gender	Age	Condition
S1	F	46	P1	M	35	SMA III
S2	F	31	P2	M	44	Muscular Dystrophy
S3	M	40	P3	M	32	Muscular Dystrophy Type II
S4	M	43	P4	F	36	Tunesian Muscular Dystrophy
S5	F	39	P5	M	25	Duchene Muscular Dystrophy
S6	M	29	P6	F	33	Tunesian Muscular Dystrophy

### Experimental environment

During the experimental procedure, participants were seated in a comfortable armchair placed 50 cm from a 22-in. Liquid Crystal Display with the EEG cap attached on their scalp. In cases where subjects used a wheelchair, appropriate modifications were made to make them feel as comfortable as possible. Throughout the entire process, subjects were instructed to place both hands in the armrests and to minimize any kind of upper limb movement in order to minimize the artifactual activity.

### Experimental design

**Fig. 1:**
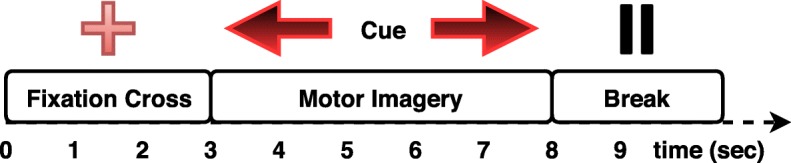
The timeline of the experimental procedure (depicted for a single-trial)

The experimental procedure required the subjects to imagine the movement of their left or right hand. Prior to the MI task, a 3 min recording of resting state was realized. The cue for the initiation of movement imagination was given by a red arrow (onset), appearing either on the left or right side of the screen, pointing in the same direction and indicating the corresponding imagery movement. The arrow remained on the screen for approximately 5 s, indicating the continuation of movement imagination to the subject. Once the arrow disappeared from the screen, subjects could rest and prepare themselves for the next arrow appearance. Prior to the arrow presentation, a fixation cross was displayed on the screen for 3 s, indicating the beginning of a new trial. Figure 1 illustrates the sequence of events during a single trial. The experimental session was divided in two sub-sessions performed during the same day, each one consisting of 20 random arrow appearances, equally distributed among the two classes, resulting in 40 trials (20 for each imagery movement class). Between the two sessions subjects had the opportunity to rest for five to 10 min. *OpenVibe,*^2^ a free and open-source platform was used to design the experimental protocol and to synchronize the EEG recording with the timestamps from the visual triggers.

### EEG recording

The brain activity was recorded, with a sampling frequency of 256 Hz, using the BePlusLTM Bioelectric Signal Amplifier,^3^ an EEG scanner with 61 + 2 (ground and reference) electrodes placed according to the 10–10 International System. Using an electro-conductive cream, the impedance for all electrodes was set bellow 10KΩ before beginning the recording in every session.

### Pre-processing

During the offline processing, the EEG signals were bandpass filtered within (0.5–45 Hz) with a third-order Butterworth filter (applied in zero-phase filtering mode), prior to the trial segmentation so as to avoid edge effects. The segmentation process resulted in 20 trials for each MI task (left and right) and 20 trials for the resting state. Using a procedure based on spectral analysis and working for each subject independently “bad” sensors were identified visually and excluded from further analysis. It is important to stress out here than on average no more than 5 sensors were rejected and the remaining (“good”) ones, denoted hereby as N_sensor_ (56 ≤ N_sensor_ ≤ 61), were employed in the subsequent average re-reference procedure. Independent component analysis (ICA) [44] was then used as a means to reduce artifacts that usually arise from eyes, muscles or cardiac pulse. Using a semi-supervised procedure that employed the ranking of independent components (ICs), based on kurtosis /skewness and the visual inspection of their spectra and topographies, artifactual components were identified and removed before reconstructing the multichannel single-trial data. For the purposes of this work, seven commonly used EEG frequency bands were defined: δ (1–4) Hz; θ (4–8) Hz; α1 (8–10) Hz; α2 (10–13) Hz; β1 (13–20) Hz; β2 (20–30) Hz; γ (30–45) Hz and the neural activity of each brain rhythm was examined independently. Once again, band-pass filtering was implemented via third-order Butterworth filters, applied in zero-phase mode.

### PLV-measurements and functional connectivity patterns

Phase synchronization is a well-established concept for describing the coordinated function of distinct neural assemblies based on the recorded signals. When studied at the level of sensor space, the brain signals recorded at distinct sites are used (by one of the available estimators) to detect whether the relative phases of the underlying oscillatory processes bear any systematic relation across time. The Phase Locking Value (PLV) measurement, introduced by Lachaux et al. [45], is a very popular estimator of phase synchrony, with the great advantage of computational simplicity that motivated its use in the context of MI-BCIs. Considered as a function, PLV gets as input two signal traces and outputs a scalar ranging between 0 and 1, with 1 indicating the functionally coupling between the brain areas associated with the signals and 0 indicating functional independence. Given a pair of single-trial signals x_k_(t) x_r_(t), with k,*r* = 1…N_sensor_ and t = t_1_… t_2_, from distinct recording sites, PLV is estimated as follows:

$$ \mathrm{PLV}\left({\mathrm{x}}_{\mathrm{k}},{\mathrm{x}}_{\mathrm{r}}\right)=\frac{1}{t_2-{t}_1}\left|\sum \limits_{t_1}^{t_2}\exp \left(\mathrm{i}\ \varDelta \upvarphi \left(\mathrm{t}\right)\right)\right| $$ (1)

with Δφ(t) = φ_k_(t)-φ_r_(t) denoting the difference between the instantaneous phases of the two processes and discrete time parameter t running along the latencies of interest (for instance the 5 s interval during the presentation of an arrow on the screen). Each phase signal φ_k_(t) is derived by applying the Hilbert transform to the corresponding band-limited brain activity x_k_(t). In our implementation, the PLV computations extend to every pair of sensors, by efficiently parallelizing the computations implied by eq.(1), as shown in Appendix. In this way, for each frequency band, an [N_sensor_ × N_sensor_] matrix is formed with entries W_kr_ = PLV(x_k_,x_r_). Adopting the popular perspective of complex networks, this matrix is treated as a weighted adjacency matrix **W** encapsulating the connectivity pattern of a graph that spans the sensor space and reflects the brain’s functional organization. Considering the symmetry in PLV measurements, PLV(x_k_,x_r_) = PLV(x_r_,x_k_) and the fact that all diagonal elements W_kk_ equal 1, it is easy to realize that a more economical description of a connectivity pattern can be obtained by vectorizing the upper triangular part of W, i.e. gathering all $\frac{N_{sensor}\times \left({N}_{sensor}-1\right)}{2}$ elements W_kr_ with *r* < k in a single vector, denoted as vec(**W**).

### Network metrics

The functional connectivity graph defined by **W** matrix, with nodes the recording sites and edges the links between the sites weighed by the associated pairwise PLV values, can be characterized based on network topology metrics [46, 47]. In our study, the network characterization was based on weighted graphs and aimed at revealing the self-organization tendencies of the underlying cortical network and contrasting them between healthy and NMD condition. Towards this end, the following three well-known metrics were estimated and compared between recording conditions (rest vs MI), as well as, physiological states (health vs NMD).

*Strength* equals to the sum of connectivity weights attached to a given node. It may serve, approximately, as a centrality measure, indicating the importance of the associated brain region within the observed network organization:

$$ {S}_k=\sum \limits_{r\ne k}{w}_{kr} $$ (2)

Global/Local Efficiency is a metric which expresses how efficiently information is transferred via the network, at a global/local level. Network’s efficiency is directly linked with the concept of *shortest paths*, which in our case were estimated after turning the functional coupling strengths w_kr_ to pairwise distances d_kr_ = 1-w_kr_ and applying the Dijkstra’s algorithm. Adopting the formulation of global efficiency (GE) as defined in (Latora and Marchiori [48]):

$$ GE=\frac{1}{N_{sensors}\left({N}_{sensors}-1\right)}\sum \limits_{k,r\ne k}\frac{1}{l_{rk}} $$ (3)

with *l*_*rk*_ denoting the length of shortest path between nodes (i.e. sensors) *r* and *k*.

Local efficiency (LE) is estimated by first restricting the above computations to each subgraph G_k_, containing the neighbors of a node k, and then integrating across nodes:

$$ \kern0.75em LE=\frac{1}{N_{sensors}}\sum \limits_{r\ne k} LE(k)=\frac{1}{N_{sensors}}\sum \limits_{r\ne k}\frac{1}{N_{G_k}\ \left({N}_{G_k}-1\right)}\ \sum \limits_{i,j\in {G}_k}\frac{1}{l_{ij}}\kern3.25em (4) $$

### Time-indexed patterns of functional connectivity

In an attempt to track more precisely the dynamics of cortical self-organization during MI, we derived multiple instantiations of the connectivity pattern for each single-trial, by means of a stepping window that confined the integration in eq. (1) within successive (overlapping) temporal segments. The width of window, T_window_, was defined according to the “cycle-criterion” (CC) [49, 50], that adapts the temporal resolution so as 3 cycles from the lowest frequency of the band-limited brain signal to be included at each step along the time-axis.^4^ In this way, a sequence vec(**W**[τ]), τ = 1,2…N_τ_ was derived that encapsulated the evolving functional connectivity during a single event of hand movement imagination. This sequence is indexed via discrete variable τ, differing from the original time variable t of the signals, to indicate that a lower temporal resolution may be utilized for reducing computational burden and memory storage. The motivation for analyzing the dynamics of connectivity patterns stemmed from previous studies, which had demonstrated that transiently formed couplings during MI [34, 35], may be useful for brain decoding.

### Feature screening

The number N_pairs_ of features corresponding to the derived PLV measurements was high. This number was ranging from 1596 to 1830, depending on the number of “bad” sensors, in the case of “static” connectivity patterns, where one vector vec([**W**]) was assigned to each single trial. This number had to be multiplied by the number of employed steps when we were dealing with time-indexed connectivity patterns (vec(**W**[τ])). This was an extremely high number of features, relatively to the small number of available trials. Apart from the theoretical issues raised by the “curse of dimensionality”, it was clear that not all possible couplings would carry highly discriminative information useful for the task of decoding left from right MI [51]. For this reason, we resorted to a “filter” approach for selecting features. Specifically, we utilized the Matlab’s *rankfeatures*^5^ command (with the option of “Wilcoxon” criterion), so as to rank the features (coupling strengths or time-resolved coupling strengths between pair of recording sites) and select the most reliable ones to participate in the subsequent design of a classifier.

More specifically, in the case of static connectivity patterns the operation of this command, denoted as follows

$$ {\displaystyle \begin{array}{l} Score(r)= rankfeatures\left(\kern0.5em {\left\{ vec\left(\  left{\boldsymbol{W}}^i\right)\right\}}_{i=1:{N}_{trials}},{\left\{ vec\left(\  right{\boldsymbol{W}}^j\right)\right\}}_{j=1:{N}_{trials}}\ \right),\\ {}r=1,2\dots {N}_{pairs}\end{array}}(5) $$

resulted in a vector of scores reflecting the relative discriminative power of each coupling. Feature selection was accomplished by identifying the set of 10 most discriminative couplings.

For the case of time-indexed connectivity, we adopted a distinct procedure that elaborated on the temporal patterning of the functional connectivity as this was unfolding during MI. The previous command was applied repeatedly at every latency τ of the stepping window resulting in a time-indexed score

$$ {\displaystyle \begin{array}{l} Score\left(r,\uptau \right)= rankfeatures\left(\ {\left\{ vec\left(\  left{\boldsymbol{W}}^i\left[\uptau \right]\right)\right\}}_{i=1:{N}_{trials}},{\left\{ vec\left(\  right{\boldsymbol{W}}^j\left[\uptau \right]\right)\right\}}_{j=1:{N}_{trials}}\ \right),\\ {}\tau =1,2\dots, {N}_{\uptau}\end{array}}(6) $$

To identify the most important features among the (N_pairs_.N_τ_) available ones, a permutation test was applied. The available connectivity patterns from “left” and “right” trials were randomly partitioned, several times, into two groups and the computations implied by eq.(7) were repeated for every random splitting. The computed {^rand^Score(r,τ)}_1:Nrand_ measurements were used to form a “baseline” distribution of scores associated with the random case, where no differences between imagination of a left and right hand movement would be detectable. From the formed distribution, the value of Score-index corresponding to the margin of 99.9% was identified and utilized as a threshold, **thr**_**99.9%**_, that was applied to the actual Score(r,τ) measurements so as to keep only the statistical significant couplings (*p*-value < 0.001). After this trimming step that zeroed most of the measurements, a sparse matrix appeared that contained some spurious entries (associated with couplings that occasionally become significant for short lasting intervals). An additional data-sieving step (based on simple rowwise median filtering) was applied that eliminated most of them. The rationale behind this last step was the detection of couplings that could be considered as both “useful” and “stable” regarding their discriminatory power. Such a reinforcement of consistency in time was motivated by the need for an economical decoding procedure and the possibility of making it functional without knowing the absolute timing (as it will be explained later). A pair-dependent profile was derived by the sequence of these operation as shown below, where the operator H(·) denotes Heaviside step function operator and **1**^**N**^ is column-vector of N ones.

$$ I\left(r,t\right)=H\left(\  Score\left(r,\uptau \right)-{\mathbf{thr}}_{\mathbf{99.9}\%}\right),\kern0.5em r=1,2,\dots \mathrm{N}\mathrm{pairs}\kern0.5em ,\mathrm{t}=1,2,\dots \mathrm{N}\uptau $$

$$ {\widehat{\boldsymbol{I}}}_{\left[\ {N}_{pairs}\times {N}_{\uptau}\ \right]}={runningMedian}_{rowwise}\ \left(\boldsymbol{I}\right) $$

$$ Profile(r)=\widehat{\boldsymbol{I}}.{\mathbf{1}}^{{\boldsymbol{N}}_{\uptau}}\kern1.5em (7) $$

**Fig. 2:**
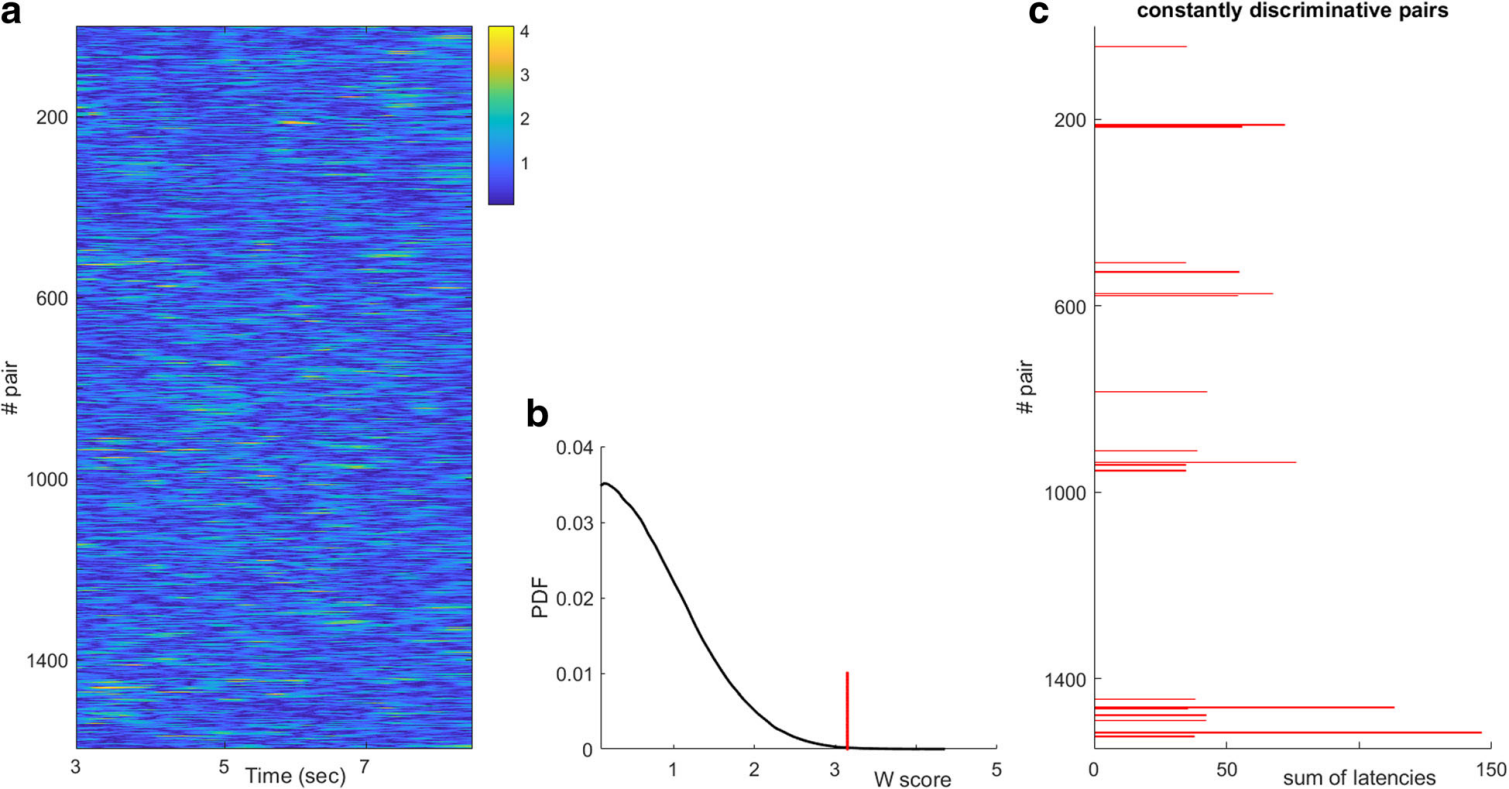
Feature Selection procedure: **a** The latency dependent Wilcoxon score for all sensor pairs. **b** The definition of a “global” threshold based on the distribution of Wilcoxon scores in randomized data. **c** The selected subset of couplings that continuously exceed this threshold for intervals longer than 100 msec (i.e. temporally consistent discriminative couplings)

Finally, feature selection was accomplished by detecting the non-zero entries in this profile. A demonstration of this sequence of algorithmic steps can be seen in Fig. 2.

### SVM-classifiers as MI-direction decoders

Support Vector Machines (SVMs) constitute a family of well-established classification algorithms [52], that is very popular among BCI practitioners [53, 54]. In the basic binary formulation, the training algorithm of SVM is designed to determine the optimal hyper-plane that separates two classes, while maximizing the margin between them. It selects the single hyperplane that guarantees optimal generalization, meaning that it can cope better with new (unseen) data. The class of an unseen pattern is determined based on its relative position with respect to the learned hyperplane, while a confidence level for this decision can be estimated by considering its distance to the hyperplane [55]. For the purposes of this study, a linear hyper-plane was selected for the MI-direction decoding as it provided satisfactory results at low computational load (a combination of high importance for online implementations). In all cases reported below, SVM classification had been employed in a “personalized” mode. This trend, that ultimately led to subject-specific brain decoding, was initiated very early during the stage of feature selection. For each trial of an MI movement, the selected discriminative features (depending on subject and brain rhythm) were used to form the input pattern to be used in SVM training and validation.

The performance of the SVM-based binary classification (“left” vs “right”) was measured, for each subject independently, under the two different feature-screening procedures, which in turn led to two distinct classification scenarios: one based on static and one based on “instantaneous” connectivity (sub)patterns. Classification performance was expressed in terms of accuracy, and carefully validated using a cross-validation scheme that was dependent on the scenario.

The validation and testing procedure was performed on a single-subject basis. In the reported results (with the exception of the results referred to self-paced MI), a leave-one-out-cross-validation (LOOCV) scheme had been employed to validate the accuracy of the proposed methodology. The use of LOOCV was motivated by the restricted amount of trials available (the sample was not big enough to employ other validation schemes like 70–30% training-test splitting of the dataset). In the LOOCV scheme, 2N_trial_-1 = 39 trials were selected as the training-set and the remaining one was used as the unknown sample that the SVM had to associate with a class. The procedure was repeated, cyclically, 40 times and the accuracy was defined based on the 40 predictions obtained from the 40 trials.

We need to clarify here that in the case of static scenario, the feature selection had been embodied in the LOOCV validation scheme (i.e. it was realized 40 times). However, this was not the case for the decoding of time-indexed connectivity patterns (vec(**W**[τ])), in which the features should show a consistency across time. In the latter case the feature selection was accomplished outside the LOOCV session of the SVM. Since the number of candidate features (pairwise couplings at multiple instances) was roughly 150 times higher than the available number of trials and therefore the danger of overfitting was even higher than in the case of static connectivity patterns we resorted to bootstrapping [56]. Having in mind to establish a procedure that could also be employed in a potential implementation of a personalized BCI, in which only a small training data-set would be available for crafting the decision function and the overall training should be completed within a reasonable time before the actual use of the BCI system, we proceeded as follows. We repeatedly form (by sampling with replacement) 30 sets of 2N_trials_, and the procedure described in eq.(7) was applied to every bootstrap-resample resulting in an ensemble of curves {^boot_i^Profile(r)}_boot_i = 1:30_. Feature selection was accomplished, by averaging these profiles and thresholding the obtained average curve.

### An SVM-ensemble for self-paced MI decoding

**Fig. 3:**
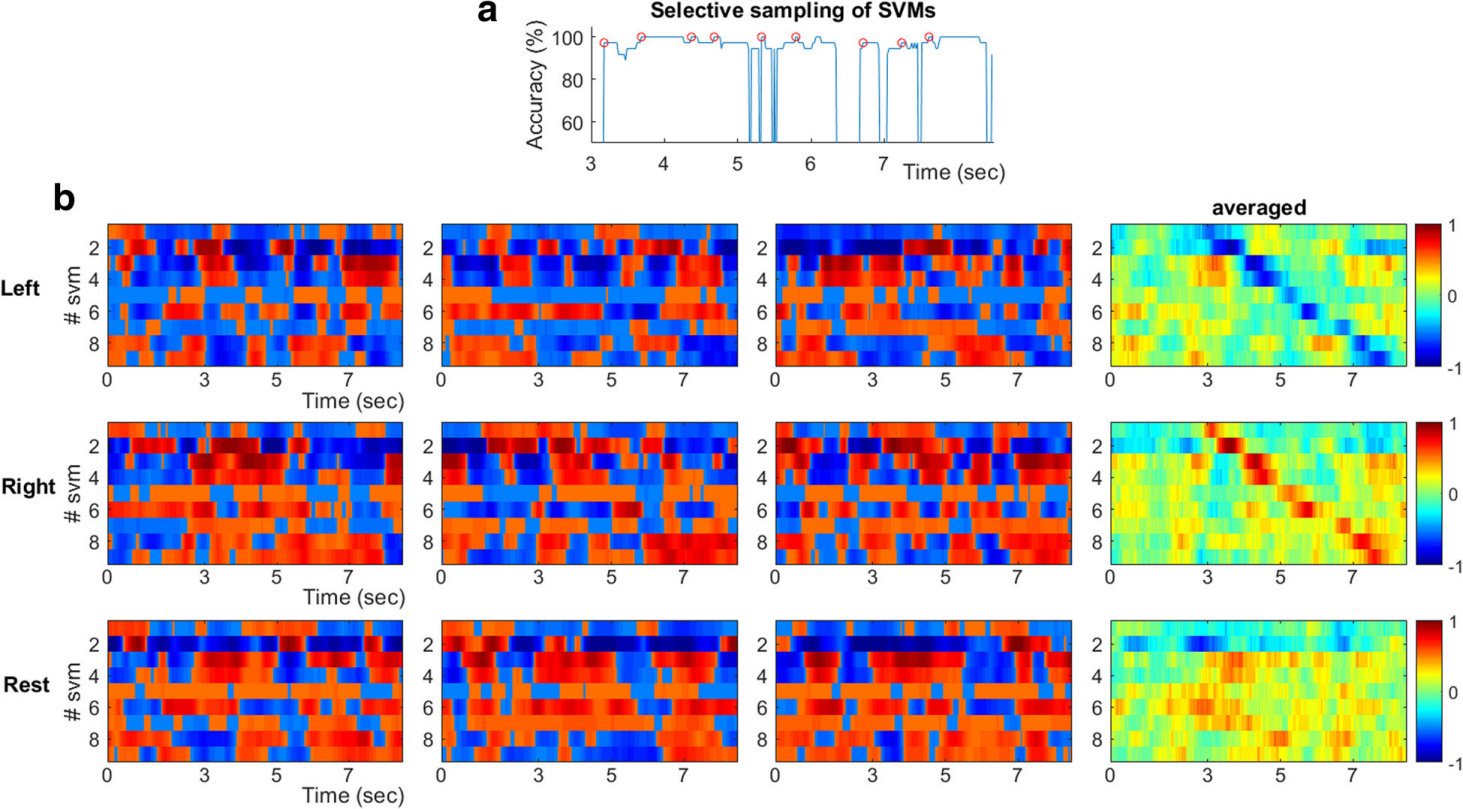
SVM-ensemble formation: **a** A set of consecutive but not “colliding” SVMs are combined in order to form an ensemble that will process, in parallel, the streaming coupling measurements and derive for each latency a vector of classification grades. **b** The latency-resolved multitude of instantaneous SVM-predictions is shown for three exemplar single trials (first three columns in every row) along with the corresponding pattern resulted from averaging the individual ST profiles across all trials (right most column). Each SVM outputs a classification score ranging within [− 1 1], with the sign indicating the movement side and the magnitude reflecting its confidence

The high performance of the SVM-decoders working with time-resolved connectivity patterns, vec(**W**[τ]), motivated us to search for a decoding scheme that could operate without the need for an external trigger that would initiate a trial. The original idea was that a “local” SVM tailored to deal with patterns from latency τ_sel_ would show a high confidence level about its prediction only within a time-interval around that latency. Adopting this consideration, connectivity patterns could continuously feed (i.e. as streaming data) to the particular SVM and its decision would be activated only whenever a certain level of confidence was reached. While this idea seemed to work well (after trial-averaging) when applied to the available MI-trials, it had the tendency to produce false-positive detections at the level of single trials (see Fig. 3b). This led us to consider not just one “time-indexed” SVM (the earliest one with the highest performance, that would satisfy the need for a speedy response), but also a sequence of them {SVM^i^}, i = τ_sel_1_,τ_sel_2_…,τ_sel_M_ with the scope of making more stringent the decision about detecting an MI event. Assuming a trigger-agnostic scenario, these SVMs will run in parallel resulting in a time-indexed vector Z(τ) = [z^1^(τ),z^2^(τ),…,z^M^(τ)]^T^, with entries

$$ {z}^i\left(\uptau \right)={\mathrm{SVM}}^i\left( FeatureExtraction\left(\ \mathrm{vec}\left(\mathbf{W}\left[\uptau \right]\right)\right)\right),i={\uptau}_{{\mathrm{sel}}_1},{\uptau}_{{\mathrm{sel}}_2},\dots .,{\uptau}_{{\mathrm{sel}}_{\mathrm{M}}}\kern2em (8) $$

Each entry z^i^ denotes the confidence of a selected classifier multiplied by the sign of its prediction (+/− is associated with “right”/“left” movement), i.e. a real number within [− 1 1]. Deviating from the standard approaches for combining classifiers (e.g. voting), in the proposed scheme the classifiers’ output are combined based on temporal patterning (that reflects their relative positioning in time, which is associated with the optimal performance in the cued trials). An “instantaneous” classification index is derived by averaging the individual signed confidences after imposing the predefined lags

$$ {z}_{\mathrm{ensemble}}\ \left(\mathrm{t}\right)=\frac{1}{M}\ {\sum}_i^M{z}^i\left(t+{\uptau}_{sel_i}\right)\kern1.25em (9) $$

It is important to note, here, that such an SVM-ensemble formation is feasible and computational tractable, thanks to the prior selection of a unique set of “stable” couplings (via bootstrapping over a small available training set). The suggested SVM-ensemble scheme is supported by two experimental observations. First, the time-indexed accuracy of the locally defined SVMs showed multiple, easy-detectable, peaks (e.g. Figure 3a). This led to an easy automation for selecting the SVMs.^6^ Second, there was no pair of SVMs among the selected “local” ones in the ensemble, that showed significant similarity.^7^ The latter fact means that all the selected SVMs were defining different separating-hyperplanes in the space of common features.

## Results

### Group analysis of pairwise couplings

**Fig. 4:**
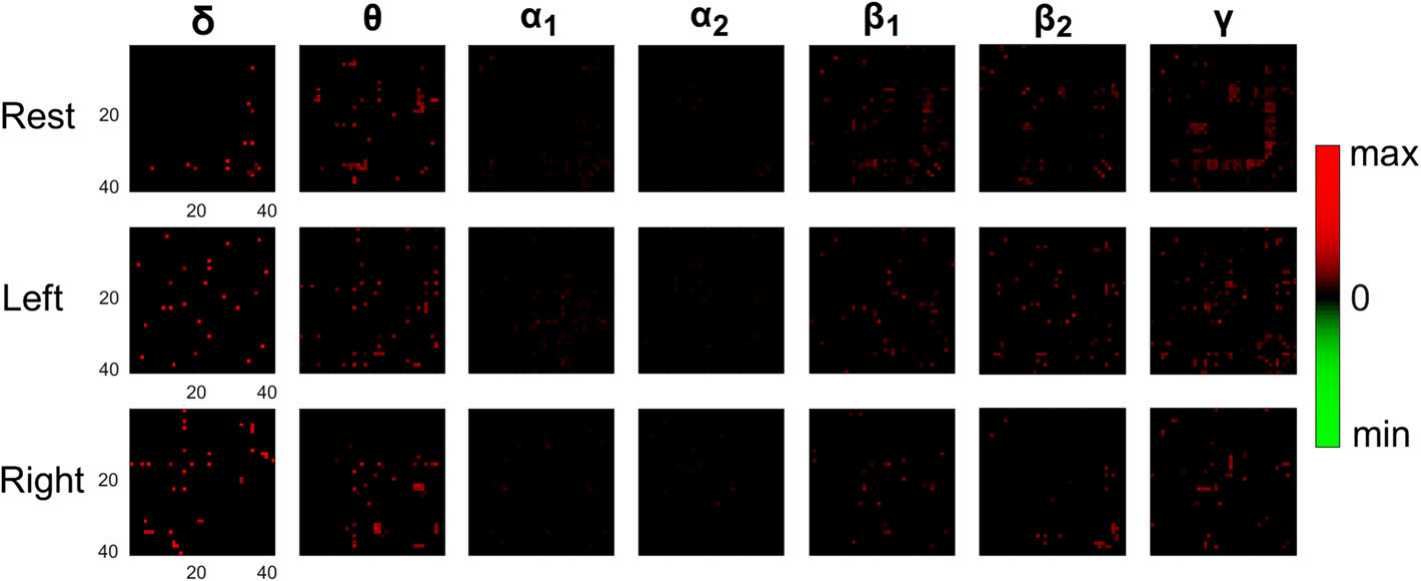
The results from the statistical comparison (Group-level analysis) of averaged connectivity patterns between patients and controls. Each pairwise coupling was compared independently, for every band and recording condition, by means of Wilcoxon rank sum and the significant ones (*p* < 0.05; corrected for multiple comparisons) are indicated as non-zeroed entries of a “connectivity matrix”, with a color code that encapsulates the difference in strength (of the median values in the corresponding groups). Red hue has to be interpreted as higher coupling in patients and green hue as higher coupling in controls, while color intensity reflects the strength of this effect. The absence of green hue in the diagrams clearly indicates the increased coupling in patients group compared to the control group

The first part of our analysis was devoted to confirming the hypothesis that there were significant differences between NMD patients and controls regarding the strength of functional couplings. To this end, a single connectivity pattern was first derived (by trial-averaging) for each subject, brain rhythm and recording condition (i.e. “rest”, “left”, “right”). To facilitate inter-subject comparisons, all the connectivity patterns were confined to the unique set of sensors that were identified as “good” sensors in all subjects. Then, a statistical comparison of the medians (derived at group level) in every pairwise coupling of the connectivity patterns was performed. The Wilcoxon rank sum test was repeatedly applied and the results were corrected for multiple testing, by means of false discovery rate (FDR; α = 0.05) [57]. Figure 4, includes the obtained results for all frequency bands and recording conditions. The statistically significant (*p* < 0.05) pairs stand out as colored entries in the shown matrices. The color in these entries reflect the sign of the observed differences. It was computed based on the medians of the groups (med({PLV(.)}^NMD^) – med({PLV(.)}^Control^)) and clearly indicates (since only red hue is observed) an increased coupling in the patients group compared to the control group, mostly in low and high brain rhythms. It is important to mention here, that increased functional couplings was found in all frequency bands, although not clearly observed when a common color code was used. The topological representation of the statistically significant functional couplings is provided in Additional file 1: Figure S1, with the edge-width reflecting the difference in strength between the group-level medians of each pairwise coupling and the node-size the number of edges that have survived the statistical test (*p* < 0.05) and are incident to the node. It is clear, that the NMD group is characterized by enhanced connectivity even in the resting state. In the two MI-conditions, the majority of nodes being part of the statistical significant couplings follow a distributed pattern, which occasionally includes the primary and supplementary sensory-motor area (for instance, in “left”: α_1_, β_1_ and γ rhythms).

### Group analysis of network metrics

**Fig. 5:**
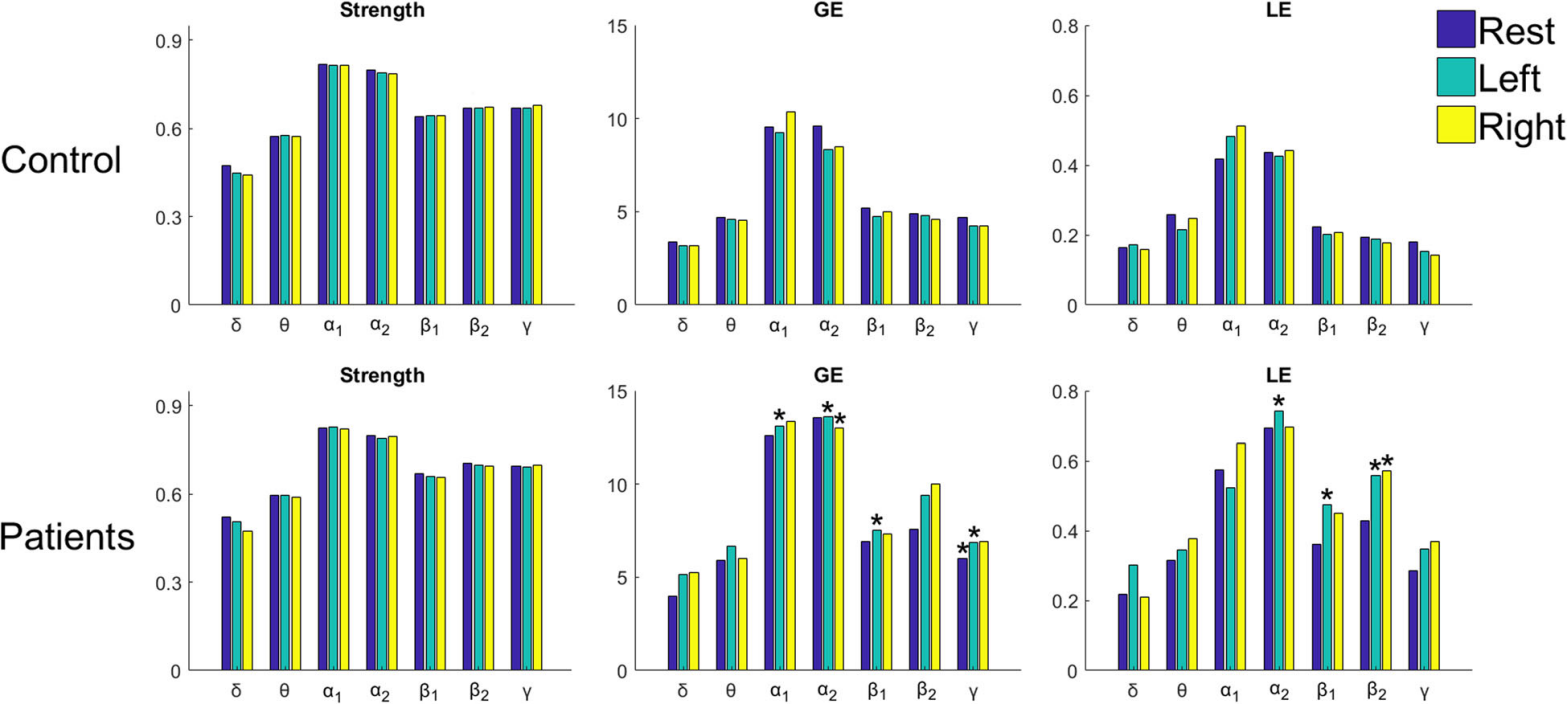
Contrasting the functional network organization between patients and controls using the standard networks metrics of *strength*, *global-efficiency* (GE) and *local efficiency* (LE). The median values, have been computed across the subjects of each group, and presented for all brain rhythms. Statistically significant differences between the two groups have also been detected (using Wilcoxon rank sum test) and indicated with a star symbol in the corresponding bar of the patients’ barplot

Next, we compared the network organization associated with the functional connectivity patterns as a means to further justify the observed differences between the two groups in terms of pairwise coupling strengths. The three metrics of Strength, GE and LE were first applied at the single-trial level (to “static” **W**s) and then averaged to derive a triad of measurements for each subject, brain rhythm and experimental condition. Figure 5 compares these measurements, after deriving group-medians. The stars in the bars of patients’ graphs indicate the statistically significant differences (*p*-value < 0.01, bonferroni-corrected) in the level of network-metrics, which resulted from the group-analysis of the corresponding measurements (NMD patients vs controls) performed using the Wilcoxon rank sum test. It is easy to observe that despite the lack of statistically significant differences in case of Strength (which practically corresponds to integrating the coupling strength across sensors), the other two metrics regarding the network’s efficiency (i.e. GE and LE) depict significant differences for rhythms faster than 8 Hz, where MI spectral activity is expected to be found. The observed differences in these two topological metrics (which reflect how efficiently the information flows within the brain network), are related to brain coordination and, hence, can be attributed to the NMD condition itself and the way it affects the patient’s brain reorganization during its progression. Interestingly, differences in network organization during MI-tasks were detected in α_2_ rhythm, even though the pairwise couplings did not show, individually, any difference between groups based on their PLV-levels (see Fig. 4 and Additional file 1: Figure S1).

### Personalized MI decoding – SVM classification based on static patterns

**Fig. 6:**
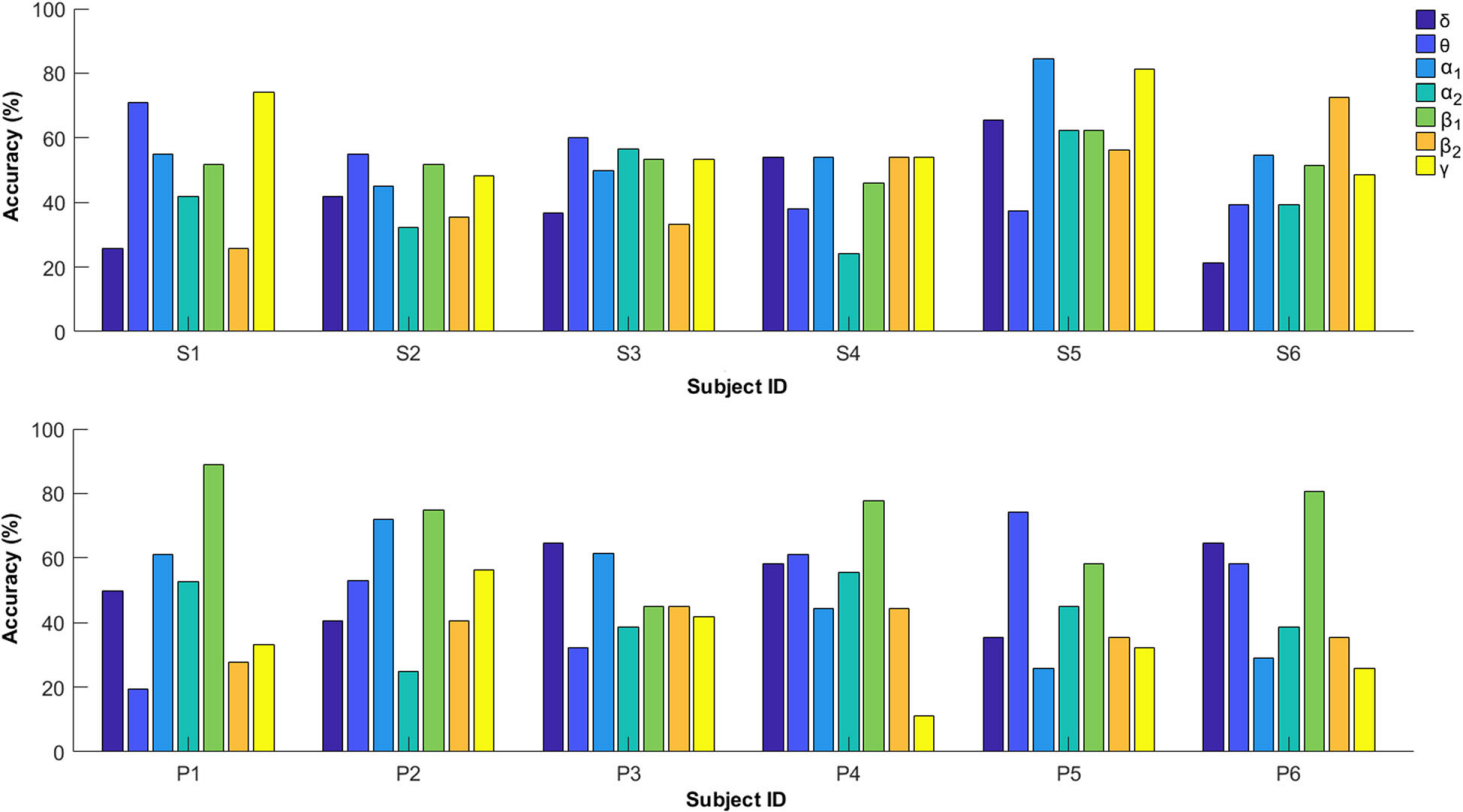
The classification performance in the state discrimination task (“left” vs “right” hand movement imagery) when elements from the static connectivity patterns are utilized

In the third stage of our analysis, we attempted to decode the MI-imagery direction based on the single-trial functional connectivity patterns and compared the performance between the two cohorts. We employed a linear SVM in conjunction with standard, statistical, feature screening. The scope of this screening was to confine the SVM design within the space spanned by the 10 most informative functional couplings. To reduce the possibility of overfitting, this feature selection step had been included in the LOOCV scheme (i.e. it was performed every time an SVM was about to be designed from the set of trials that had been reserved for training). The classification accuracy of the “left vs. right” decoding task for each subject and brain rhythm is shown in Fig. 6, where it can be justified that working at a personalized level was indeed necessary, since performance (and frequency-band of optimal performance) varied a lot across subjects. It is evident that the accuracy levels for the patients group are significantly higher, with five out of six subjects exceeding 75% accuracy and even the subject with the lowest accuracy (i.e. P3) for this group reaches 65%. It is also interesting to notice that for the NMD group the highest accuracy is associated with β1 (13–20 Hz) band in four subjects (i.e. P1, P2, P4, P6). On the other hand, half of the control subjects do not surpass the level of 60% accuracy in any of the frequency bands, with subject S5 standing as the best subject for the control group, as it is the only case were 80% of the trials were correctly classified. To confirm rigorously the hypothesis that BCI-naïve patients can perform better than controls in the employed MI tasks, we gathered the highest performance level from each individual in two sets of accuracies, {^NMD^Accuracies}_*i* = 1:6_ / {^controls^Accuracies}_i = 1:6_, and applied the Wilcoxon rank sum test that revealed a statistical significant difference (*p* < 0.05, one-tailed).

For comparison purposes we have included, as Additional file 1: Figure S2, the results from decoding MI-direction based on power spectral density (PSD) estimates, where the feature screening was applied to the ensemble of PSD measurements (that included the measurements from every sensor and brain rhythm). Overall, the decoding performance stays below 75% (except for patient P5), i.e. lower than in the case of PLV measurements. In addition, there is no statistically significant advantage for the NMD group over the controls considering the highest performance level from each individual (*p* = 0.43, one-tailed). Similar trends were obtained from a decoding scheme based on CSPs (see Additional file 1: Figure S3).

### Personalized MI decoding – SVM classification based on time-varying patterns

**Fig. 7:**
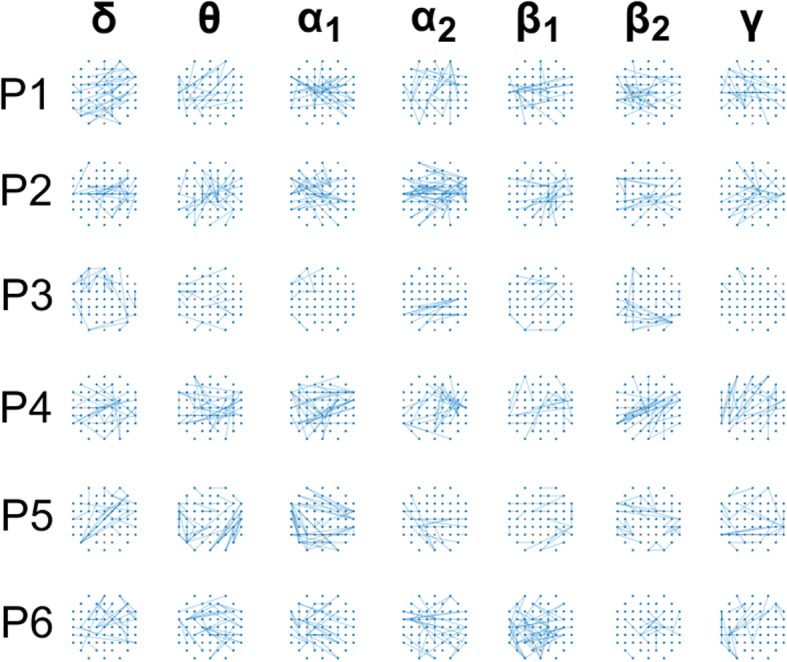
The statistically significant and temporally consistent couplings as detected by means of a permutation test (random re-labeling of trials), applied for each patient and brain rhythm independently

At the expense of increased computations and algorithmic complexity, we then moved to decoding MI-direction from time-varying connectivity patterns for the NMD-patients. Both the beneficial phase-synchrony based representation, for the brain activity in this clinical group, and the fact that MI-BCIs have remained largely unexplored for NMD patients led us to study deeper the relevant dynamic patterns of connectivity. Supporting evidence, regarding the dynamic nature of the underlying phenomena, was offered by the feature screening procedures, since the scores acquired by dynamic patterns were often higher than the ones obtained from static patterns.^8^ Working at a personalized level, we first identify the set of functional couplings that showed a stable and highly discriminative behavior (using bootstrapping and eq.(7)). These couplings have been in included in Fig. 7. The fixed set of selected entries were extracted, in every single-trial, from the time-indexed connectivity patterns, vec(**W**[τ]), which had been computed with a time-step of 350 ms. The vectors were used to design and evaluate an “instantaneous” SVM (i.e. SVM^τ^) that corresponds to each latency and also follows a LOOCV scheme. The performances of this decoder were estimated by comparing the time-indexed predictions with the class labels of the trials and integrating the results across trials.

**Fig. 8:**
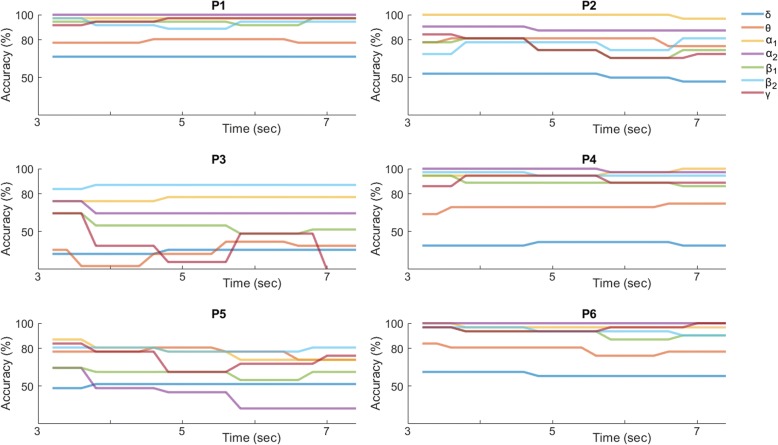
The classification performance in the “left” vs “right” task, when the selected couplings (shown in Fig. 7) are used to form multiple time-resolved patterns associated with each trial

Figure 8 shows the corresponding performance curves for the “instantaneous” SVM classification scheme. At every latency the performance was estimated based on the selected couplings (shown in Fig. 7). It is clear, that there is variability among subjects. There are subjects (P1, P3 and P6) reaching the highest accuracy within the first second and maintaining the high performance for the full trial length. On the other hand, subjects P2 and P4 do as well achieve the highest performance levels within the first second but do not maintain it for the trial’s full length. Such a trend could be interpreted as declining engagement to the task. Finally, one subject (P5) showed deterioration in performance after the first second. The observed variability can be attributed to the subject’s devotion to the task, how he/she performed it, and possibly to the type of NMD. Overall, this classification scheme appears to lead to optimal performance earlier in time.

### In quest of self-paced MI decoding

**Fig. 9:**
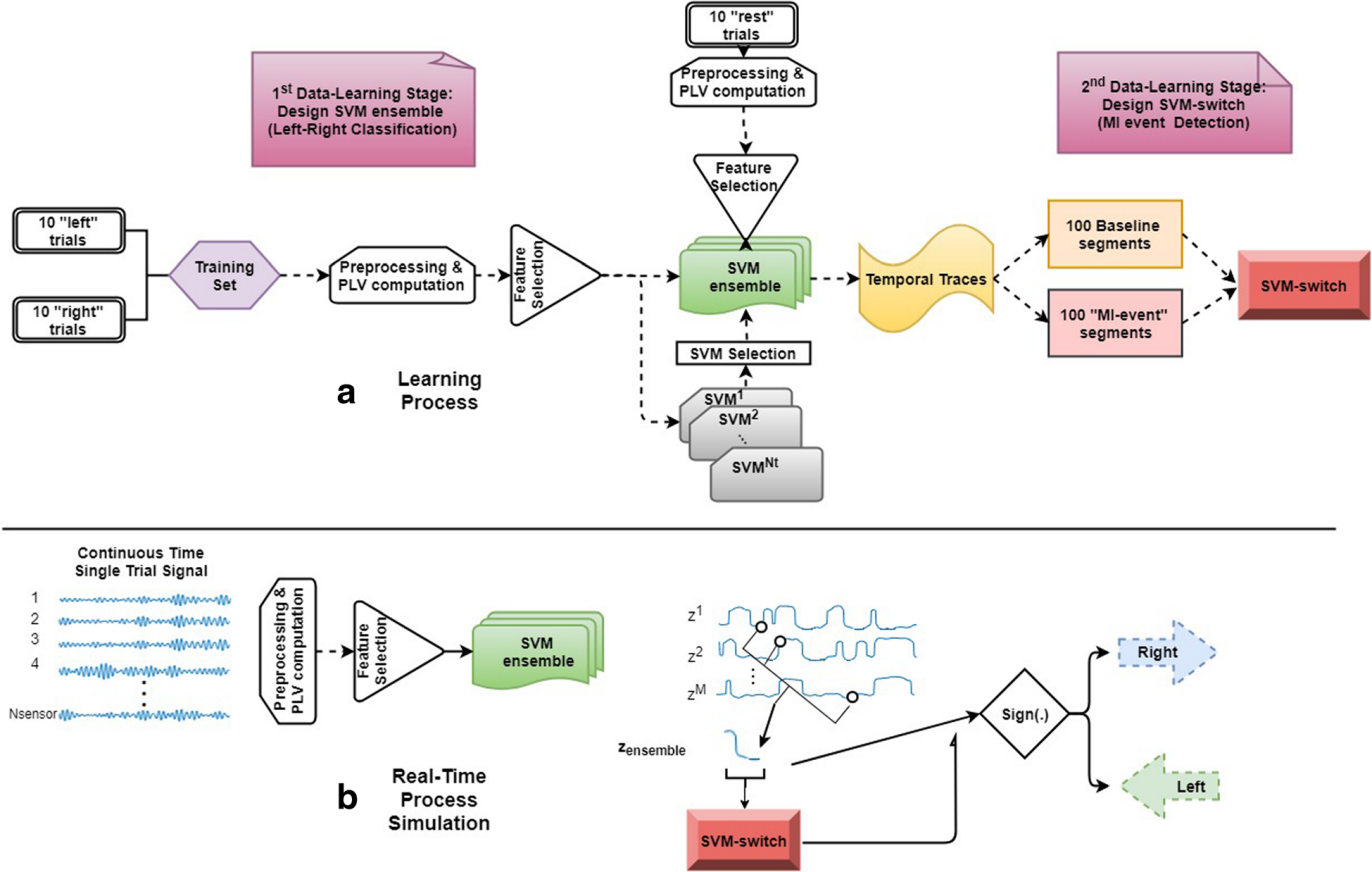
**a** Flowchart of the data leaning process for the self-paced MI decoding. **b** Graphical depiction of the decoding procedure from the streaming connectivity patterns during one single trial

Finally, we explored the possibility of decoding phase-connectivity patterns in a way that could be used in a future implementation of a self-paced MI-BCI, where the user would initiate the MI events at will. Since there were no recordings of self-initiated MI events, we decided to partially “simulate” the case by exploiting the resting-condition recordings and devising a scheme that would mark a time instance as the beginning of an MI-event (“left” or “right”) only when the temporal patterning in the streaming connectivity-data was deviant from the patterning in the baseline (rest) condition. To this end, 20 trials were extracted from each patient’s resting-state recording and “baseline” time-resolved connectivity patterns (extending for 8 s) were formed, based on the same signal-analytic pipeline used in the case of MI-trials. Since the purpose of this analysis was the reliable detection of dynamical transitions in brain state (from “idle” to an MI event), the connectivity patterns from resting state and, also, the MI events were derived with high temporal resolution (based on a step of 20 msec), so as to emphasize the temporal aspects of the detection task. To ease the presentation of the results reported in this section, Fig. 9 depicts graphically the employed algorithmic steps.

A training set consisting of 10 trials from each class (“left”, “right” and “rest”) was formed and utilized in a two-stage data-learning process. During the first-stage, only the MI-related single-trial connectivity patterns ({^left^vec(**W**^i^[τ]}_*i* = 1:10_ and {^right^vec(**W**^i^[τ]}_i = 1:10_) were used for a) the feature-selection, b) the training of all “instantaneous” SVMs, c) the selection among them, of those that populated the ensemble {SVM^i^}. The feature selection step is exemplified in Fig. 2, for subject P2’s connectivity patterns from α_1_ rhythm. The selection of SVMs is exemplified in Fig. 3a, while the application of the SVM-ensemble in some trials (from all recording conditions) is demonstrated in Fig. 3b, where the vectors of successive predictions appear as columns in the shown heat-maps. The right-most panels in Fig. 3b includes the corresponding trial-averaged heat-maps, where a “diagonal” pattern is emerging in both cases of “triggered” MI-events but not in the case of resting-state. It was exactly this discrepancy, that the stratified combination of the outputs of the SVMs participating in the ensemble, tried to reveal, in a computationally tractable way, by means of eq.(9).

**Fig. 10:**
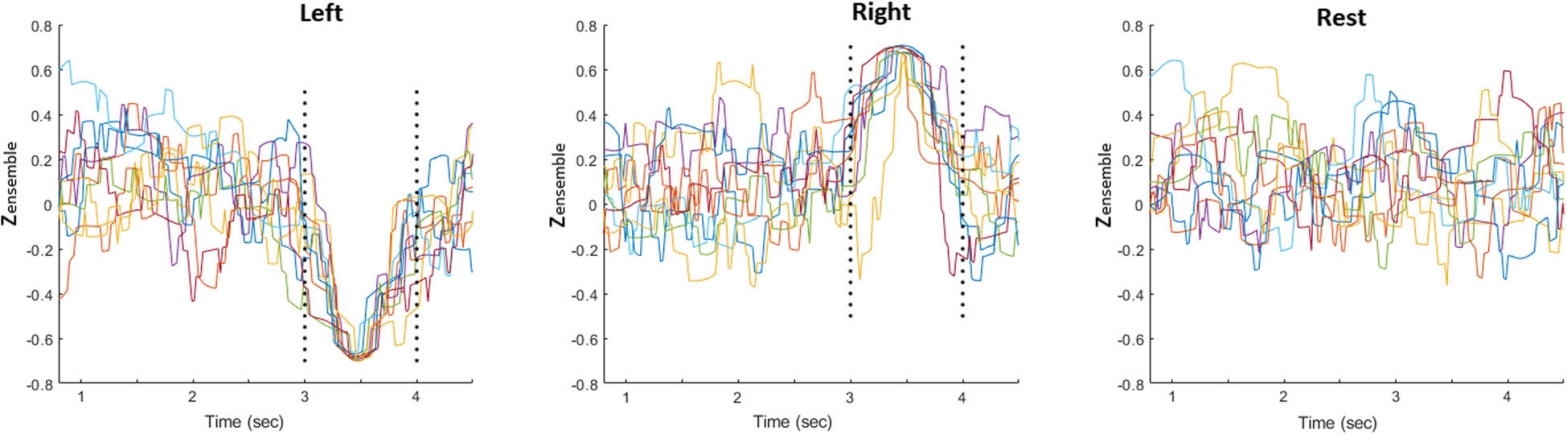
The latency-dependent classification-index Z_ensemble_ as derived, by means of the time-lagged combination of the SVM-ensemble readouts, for a training set of trials

**Fig. 11:**
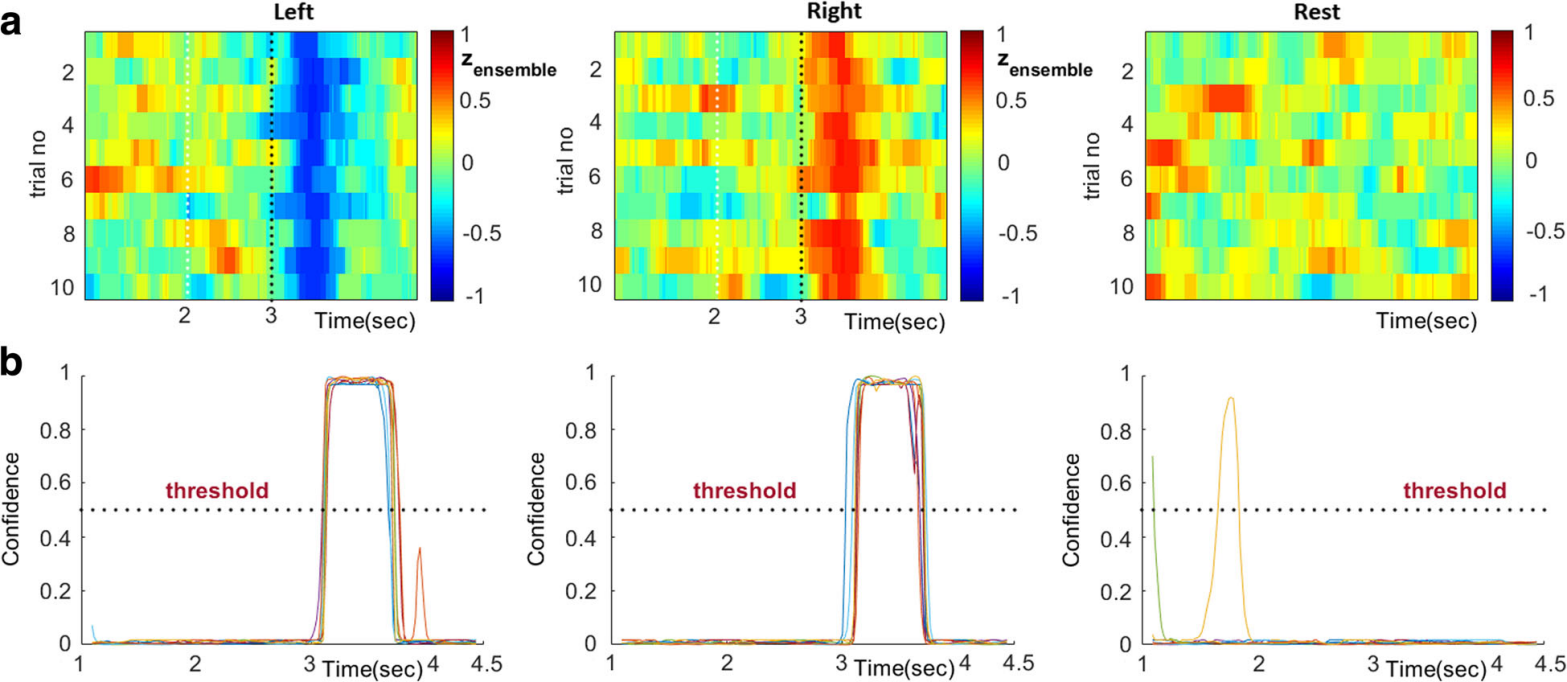
**a** The latency-resolved multitude of instantaneous SVM-predictions is shown for a test-set trials. **b** The latency-dependent confidence of the SVM-decider (trained based on temporal patterns extracted from Fig. 10)

During the second stage, the temporal traces corresponding to the single-trial “instantaneous” readouts from the SVM-ensemble were derived for the above mentioned MI-related connectivity patterns and, in addition, for the baseline-related ones {^rest^vec(**W**^i^[τ]}_*i* = 1:10_. Figure 10 demonstrates the estimated traces of Classification Index, z_ensemble_(t), in continuation of the example shown previously in Figs. 2 and 3. It is evident that a peak is identifiable, just after the 3rd second (onset), for both the “left” and “right” conditions. On the contrary, the traces derived from the rest condition trials do not illustrate any comparable peak. In an attempt to quantify these observations, and simultaneously complete the design of a totally self-paced MI-decoding scheme, we used these 30 profiles (as training data) to craft a decision rule, that based on streaming data (a segment of SVM-ensemble readout) would decide if the observed temporal patterning in Classification Index corresponded to baseline condition or to an MI event and, hence, should trigger the command associated with the sign of the trace from the SVM-ensemble. To accomplish the data-learning task, we extracted multiple segments, of 0.5 s width, from the singe-trial traces shown in Fig. 10 and confined within the intervals indicated via vertical dotted lines. These 100 segments were corresponding to the “MI-event” class (regardless direction). An equal number of segments were extracted from the baseline condition, but this time without any restriction about the time interval. These segments constituted the “baseline” class patterns. Both type of segments were used for training a binary-SVM (with a radial basis function kernel) to discriminate an MI event from the baseline state. The trained “SVM-switch”, was then fed with the streaming SVM-ensemble readouts, z_ensemble_(t), resulted from the testing set of trials. Figure 11, exemplifies this step by first depicting the “instantaneous” single-trial readouts form the SVM-ensemble (formed in Fig. 3a) for the three recording conditions (Fig. 11a) and, then, the corresponding single-trial traces of the instantaneous confidence of the SVM-switch (all the consecutive segments had been fed to this classifier) (Fig. 11b). Using as threshold, the confidence level of 0.5, we obtained only 2 false positive (FP) detections in all three recording conditions (please notice that this would had also been the case if a high confidence level had appeared within the first 3 s interval of a MI trial), and no false negative ones. After referencing these counts to the number of trials, we estimated two probabilistic indices regarding the observed probabilities of FP and FN (here 2/30 and 0/30 respectively).

The overall procedure was repeated after different randomized partitions of the data (i.e. Monte-Carlo cross validation scheme), and the results (after averaging across 100 splits) were tabulated in Table 2. The brain rhythms had been selected according to the performance levels shown in Fig. 8.

**Table 2:** FP/FN for the SVM-switch

Participant ID	P1	P2	P3	P4	P5	P6
brain rhythm	α_2_	α_1_	β_2_	α_2_	α_1_	α_2_
FP	2.2%	3.5%	7.2%	3.9%	7.2%	2.7%
FN	1.4%	2.0%	5.3%	2.1%	3.3%	1.4%

The very low probabilities of misdetection and false alarm, in conjunction with the very high performance of the individual MI-decoders participating in the ensemble, make the combined scheme (SVM-ensemble & SVM-switch) potentially suitable for self-paced MI-decoding (see Fig. 9).

## Discussion

NMD is a condition that gradually affects the musculature and eventually leads to the loss of any voluntary muscle control. The reflections of NMD on the electroencephalographic brain activity, under the perspective of establishing efficient BCIs, have rarely been studied [42]. It was the scope of this study to examine the differences in the functional brain organization between NMD patients and healthy individuals in a motor-imagery paradigm that, traditionally, is considered fruitful for endogenous BCIs. Rhythm-specific connectivity patterns during motor imagery and resting state were derived and used, first, to contrast the two cohorts in terms of coupling strength and network organization and, then, to explore different possibilities for MI-event decoding and detection schemes, in NMD patients. Special attention was paid to dynamic patterns of functional connectivity in an attempt to identify faster ways to perform MI decoding and relax the dependence of this decoding from external triggering.

Overall, the reported results provide empirical evidence about the hypothesis that NMD patients could perform well in MI tasks, without any training, due to the equivalence, for them, of performing an imagery movement and an actual one; or, equivalently, due to the fact that the disease’s progression simulates a long training phase. More specifically, the pairwise phase-coupling was found statistically elevated in NMD patients (Fig. 4 and Additional file 1: Figure S1) and the network organization (associated with faster rhythms) significantly higher (Fig. 5). In addition, MI-decoding, worked out in a personalized manner, was performed more efficiently in patients than in controls (Fig. 6). It is important to notice that Phase-synchrony representation resulted in a more reliable decoding than signal-power representation (compare Fig. 6 with Additional file 1: Figure S2) and CSP approach (Additional file 1: Figure S3).

Moreover, our results also showed that direction decoding can be performed, almost equally well, by training time-indexed SVM-decoders using phase synchrony patterns that are regularly sampled from the post-stimulus time interval (Fig. 8) opening the possibility of reducing the response time in cued MI-based BCIs. This observation led to a lagged combination of distinct SVM-decoders that all operated in the same feature space (Fig. 2) but trained with different time-indexed instantiations of the training set of phase synchrony patterns (Fig. 3a). The introduced combination of SVM-activations acts as an optimal filter that can run in real-time and reliably trigger the recognition of an MI-event (Fig. 11), the direction of which is conveyed by the polarity of the assembled classification-index.

The importance of this work stems from fact that (to the best of our knowledge) there is only one paper that tackles the same problem that is MI-BCI for NMD patients [42]. In line with our work, the authors demonstrate the successful use of BCI. However, MI-decoding is based on time-domain characteristics and requires significant amount of training time (8–12 training sessions).

There are some novelty aspects in this work, that need to be put in the context of contemporary practice in neuroscience research and streaming data analysis. First, we need to underline our choice to work with dynamic phase synchrony patterns, casting new empirical evidence about the benefits of *chronnectomics* (“chronos” = time + “connectomics”), an emerging branch of network neuroscience that focuses on the dynamics of brain-network (self)organization phenomena [58–61]. Phase locking computations can be implemented efficiently from multisite recordings, as already has been pointed out by a recent work [62] and indicated in the Appendix. This computational efficiency, together with the fact that the MI-related network reorganizations are characterized by fast transitions, opens the possibility of prompt MI-detection and decoding in nearly real-time. The second point that deserves further consideration is the SVM-ensemble formation and its use in filtering mode (i.e. its application to streaming connectivity patterns). While such an implementation of SVMs seems rather unusual in EEG-related research, it has already been successfully employed for continuous speech recognition (for instance [63, 64]).

Finally, the main limitations of this study need to be discussed, starting with the restricted number of available trials. Even though precautions were taken (by means of cross-validation) to avoid overfitting, our findings will wait the verification from further studies. Particularly the self-paced MI-decoding scheme was demonstrated and validated using a “crude simulation”. This part of our study needs to be treated strictly as a proof-of-concept, since trials from an independent resting state recording were treated as extracts from continuous data interrupted by MI-events. Secondly, although it is common practice in studies with people with disabilities to include a restrained number of participants [35–37, 40], as the recruitment process is not as straightforward as in control population, it would be of great importance to further validate the statistical differences between the groups by encountering higher number of NMD participants in the MI experimental procedure. Thirdly, all the reported results were obtained from off-line analysis, in which “cleaned” data were employed (see Pre-processing section). It remains to show that (whether) the proposed decoding scheme is robust to artifacts like blinks; a possibility that rises since it revolves around phase-descriptor. Alternatively, in a realistic implementation one of the available real-time artifact-removal techniques may be incorporated [65, 66]. Therefore, the evaluation regarding the methodology’s performance in terms of challenging, real-time conditions is yet to be explored and is considered to be an intriguing part of any future actions aiming to “build” a self-paced MI BCI. Moreover, a personalized (subject-adaptive) data-learning scheme was pursued for the purpose of MI-decoding. This, inevitably, makes necessary a small training set before a participant can take advantage of the suggested MI-decoding mechanism. While principles of transfer learning maybe useful, we tend to consider as best practice a small training session in which self-initiated MI-events will be embedded in a “relevant” baseline activity recoding (for instance, watching a videoclips sequence and “instruct” skipping the current one by imagining a hand movement). Finally, connectivity patterns were estimated at sensor level and the issue of volume conduction was not addressed since favorable results were obtained readily and the precise modelling was considered beyond the scope of MI-BCIs.

## Conclusions

NMD patients appear to possess an inherent advantage, over healthy subjects, in the use of phase-synchrony related MI-BCIs. Patient-specific data-learning procedures have the potential for leading to effective brain decoding schemes from the emerging connectivity patterns, that can be implemented efficiently and, when embedded in patient’s daily life, provide a certain level of autonomy.

### Additional file

Additional file 1: **Figure S1.** Topographical representation of the statistically significant functional couplings (shown in Fig. 4). In the emerging graphs, the edge-width reflects the strength of the coupling and the node-size the number of edges incident to that node. The shown results correspond to Group-level analysis and reflect higher connectivity in the NMD patients. **Figure S2.** The classification performance in the state discrimination task (“left” vs “right”), when band-specific power-spectral density estimates are employed. **Figure S3.** The classification performance in the state discrimination task (“left” vs “right”), when the Common Spatial Pattern algorithm is employed in the 8–30 Hz frequency band as described by Fabien Lotte [1]. (ZIP 819 kb)

## Appendix

In the following Matlab-coded implementation of the PLV-computations, the function receives as input the multichannel-signal matrix (row-vectors correspond to sensors) and outputs the square matrix **W** containing all pairwise couplings

function W=Fast_PLV_for_multichannel_signal(filtered_traces)

% PLV_Matrix = Fast_PLV_for_multichannel_signal(filtered_traces)

% filtered_traces: [N_sensors x N_timepoints] matrix of band-limited signals

% PLV_Matrix: [N_sensors x N_sensors] matrix of pairwise PLVs

[Nsensors,Ntime] = size(filtered_traces);

Phases = angle(hilbert(filtered_signals’))’; Q = (exp(j*Phases));

W = (1/Ntime)*abs(Q * Q’)

## Abbreviations

ALS: Amyotrophic Lateral Sclerosis
BCI: Brain Computer Interface
CS: Chronic Strokes
CSP: Common Spatial Patterns
EEG: Electroencephalography
ERD/ERS: Event Related Desynchronization/ Event Related Desynchronization
ERP: Event Related Potentials
FDR: False Discovery Rate
FN: False Negative
FP: False Positive
GE: Global Efficiency
ICA: Independent Component Analysis
LE: Local Efficiency
LOOCV: Leave One Out Cross Validation
MI: Motor Imagery
MS: Multiple Sclerosis
NMD: Neuromuscular Disease
PLV: Phase Locking Value
SCI: Spinal Cord Injury
SMR: Sensorimotor Rhythm
SVM: Support Vector Machines
WHO: World Health Organization

## Footnotes

1
http://www.who.int/disabilities/world_report/2011/report/en/

2 http://openvibe.inria.fr/

3
http://www.ebneuro.biz/en/neurology/ebneuro/galileo-suite/be-plus-ltm

4 For instance, in the case of δ band ([f_1_, f_2_] = [1, 4] Hz), and since the sampling frequency was f_s_ = 256 Hz, the size of window was ${T}_{window}=\frac{CC}{f_1}\times {f}_s=\frac{3}{1}\times 256=768\kern0.5em samples$.

5
https://www.mathworks.com/help/bioinfo/ref/rankfeatures.html

6
https://www.mathworks.com/help/signal/ref/findpeaks.html

7 The correlation coefficient ρ(z^i^(t),z^j^(t)), i,j = 1…M, averaged across trials, was lower than 0.2.

8 i.e. *max*_*r*, *τ*_(*Score*(*r*, *τ*) > *max*_*r*_(*Score*(*τ*)) following the notation of eq.(5) and eq.(6)).
