# Supplementary material for: Exploiting the heightened phase synchrony in patients with neuromuscular disease for the establishment of efficient motor imagery BCIs
Source: J Neuroeng Rehabil. 2018 Oct 29;15:90. doi: 10.1186/s12984-018-0431-6 (PMC6206934; doi:10.1186/s12984-018-0431-6)
Supplement: Supplementary file 1 — Figure S1. Topographical representation of the statistically significant functional couplings (shown in Fig. 4). In the emerging graphs, the edge-width reflects the strength of the coupling and the node-size the number of edges incident to that node. The shown results correspond to Group-level analysis and reflect higher connectivity in the NMD patients. Figure S2. The classification performance in the state discrimination task (“left” vs “right”), when band-specific power-spectral density estimates are employed. Figure S3. The classification performance in the state discrimination task (“left” vs “right”), when the Common Spatial Pattern algorithm is employed in the 8–30 Hz frequency band as described by Fabien Lotte [1]. (ZIP 819 kb) [file 12984_2018_431_MOESM1_ESM.zip › supplement_Georgiadis_JNER-D-18-00104_HL.docx]

**Additional file 1**

Exploiting the heightened phase synchrony in patients with neuromuscular disease for the establishment of efficient motor imagery BCIs

Kostas Georgiadis^* 1, 2^, Nikos Laskaris^1, 3^, Spiros Nikolopoulos^2^ and Ioannis Kompatsiaris^2^

Address: ^1^ AIIA lab, Informatics dept., AUTH, Thessaloniki, Greece, ^2^ Information Technologies Institute (ITI), Centre for Research & Technology Hellas, Thessaloniki-Thermi Greece and

^3^ NeuroInformatics.GRoup, AUTH, Thessaloniki, Greece

E-mail: Kostas Georgiadis - georgiaki@csd.auth.gr; Nikos Laskaris - laskaris@aiia.csd.auth.gr; Spiros Nikolopoulos - nikolopo@iti.gr; Ioannis Kompatsiaris - ikom@iti.gr

^*^ Corresponding author


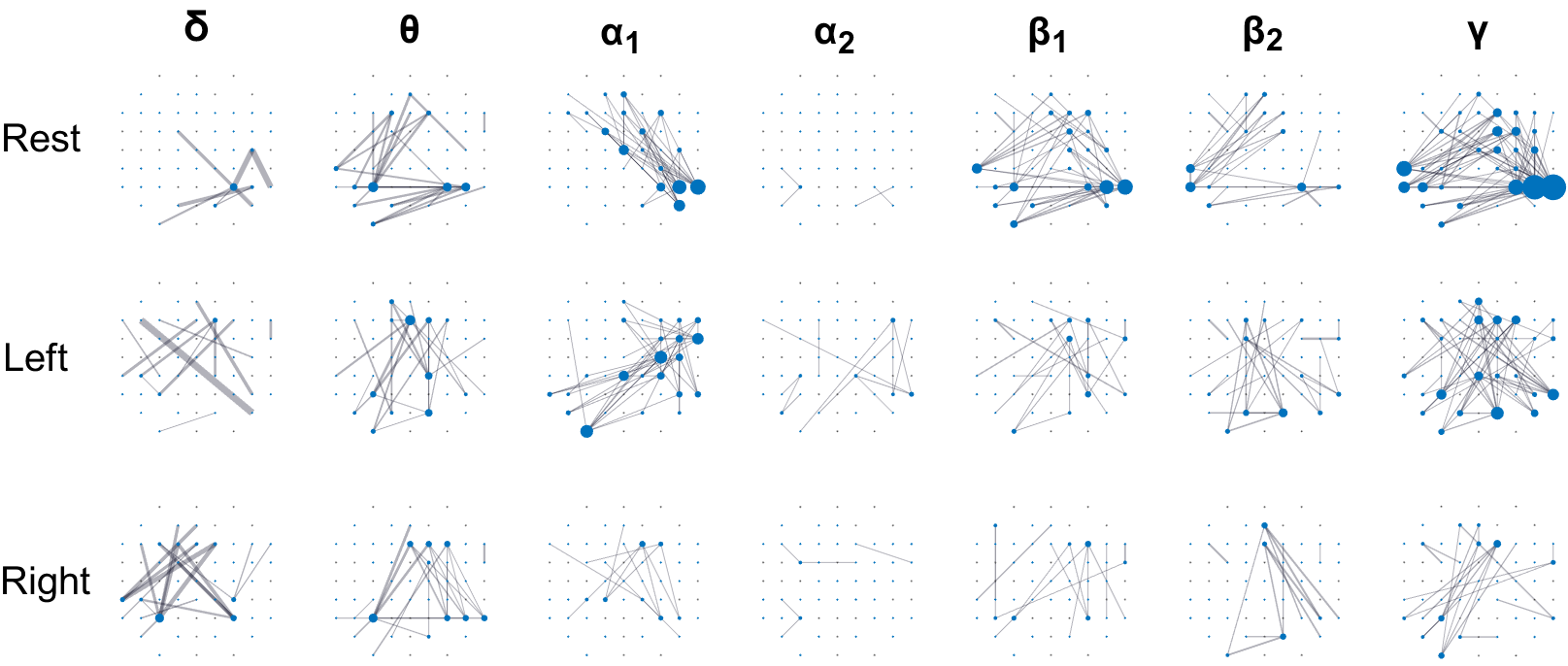


**Figure S1.** Topographical representation of the statistically significant functional couplings (shown in Fig.2). In the emerging graphs, the edge-width reflects the strength of the coupling and the node-size the number of edges incident to that node. The shown results correspond to Group-level analysis and reflect higher connectivity in the NMD patients.


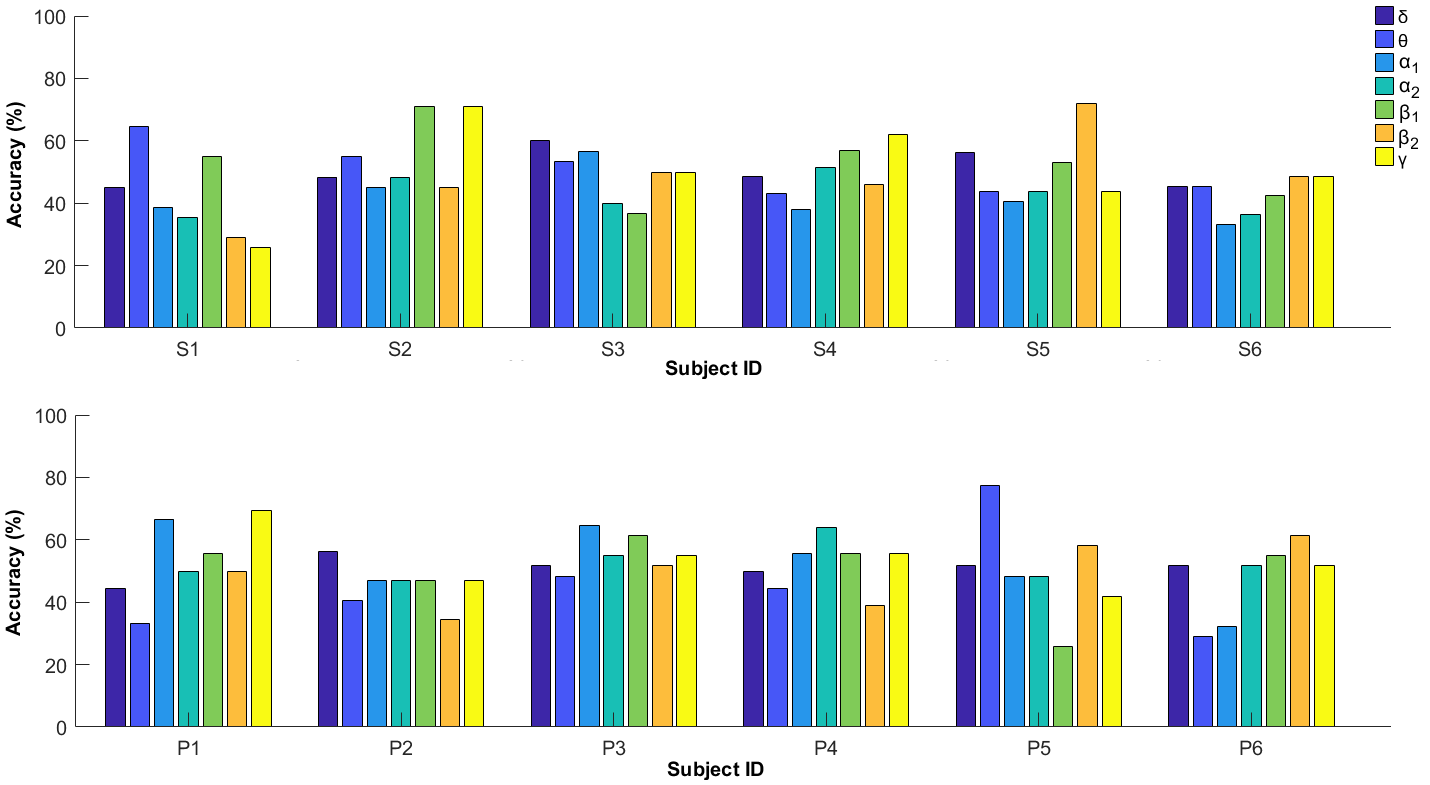


**Figure S2.** The classification performance in the state discrimination task (“left” vs “right”), when band-specific power-spectral density estimates are employed.


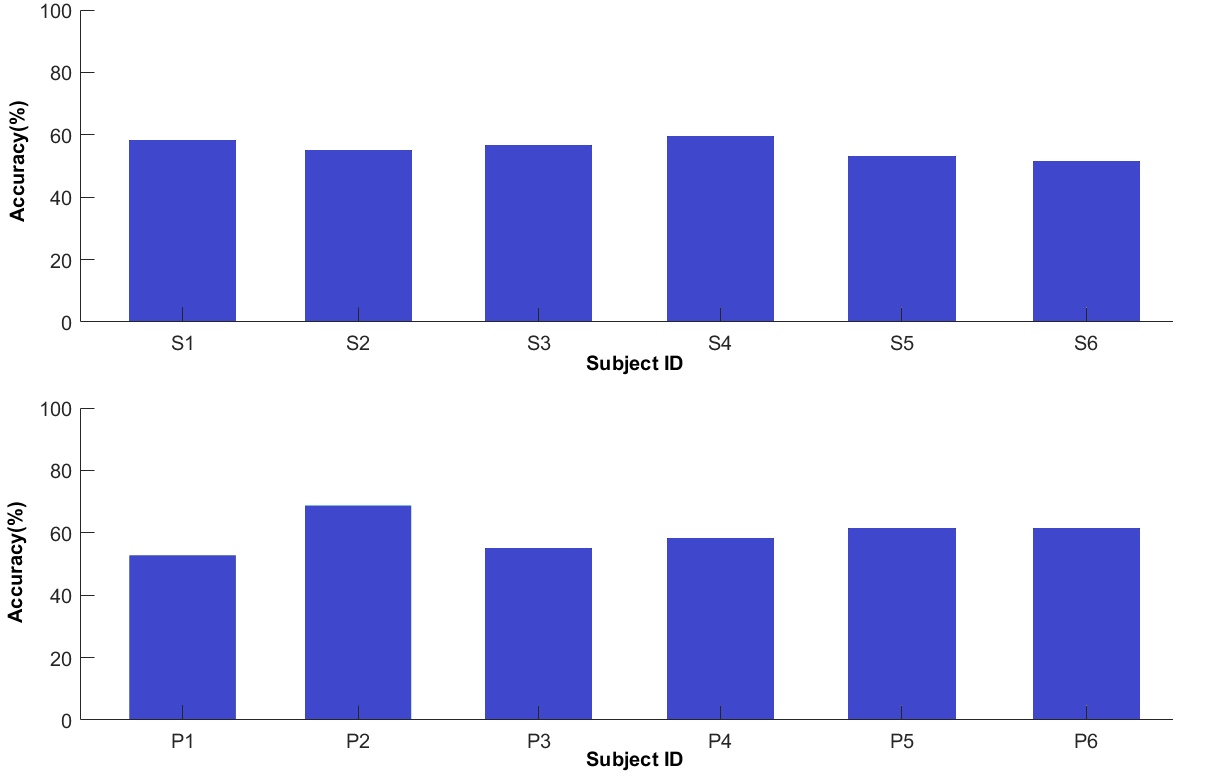


**Figure S3.** The classification performance in the state discrimination task (“left” vs “right”), when the Common Spatial Pattern algorithm is employed in the 8-30 Hz frequency band as described by Fabien Lotte [1].

[1] Lotte F. Signal processing approaches to minimize or suppress calibration time in oscillatory activity-based brain–computer interfaces. Proceedings of the IEEE. 2015 Jun;103(6):871-90.
