# Supplementary figures and images for: Exploiting the heightened phase synchrony in patients with neuromuscular disease for the establishment of efficient motor imagery BCIs
Source: J Neuroeng Rehabil. 2018 Oct 29;15:90. doi: 10.1186/s12984-018-0431-6 (PMC6206934; doi:10.1186/s12984-018-0431-6)

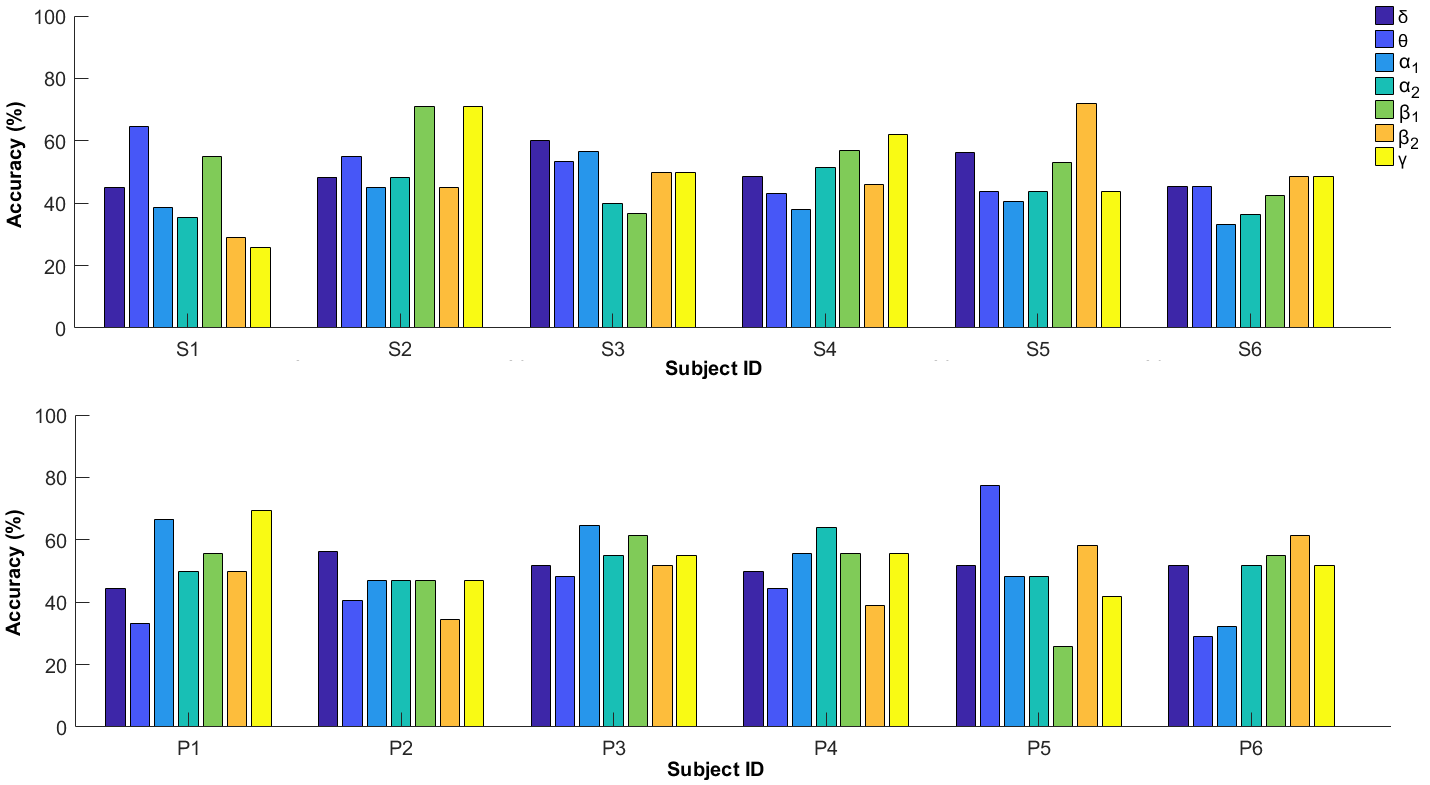

Supplement: Supplementary file 1 — Figure S1. Topographical representation of the statistically significant functional couplings (shown in Fig. 4). In the emerging graphs, the edge-width reflects the strength of the coupling and the node-size the number of edges incident to that node. The shown results correspond to Group-level analysis and reflect higher connectivity in the NMD patients. Figure S2. The classification performance in the state discrimination task (“left” vs “right”), when band-specific power-spectral density estimates are employed. Figure S3. The classification performance in the state discrimination task (“left” vs “right”), when the Common Spatial Pattern algorithm is employed in the 8–30 Hz frequency band as described by Fabien Lotte [1]. (ZIP 819 kb) [file 12984_2018_431_MOESM1_ESM.zip › Figure_S2.tif]

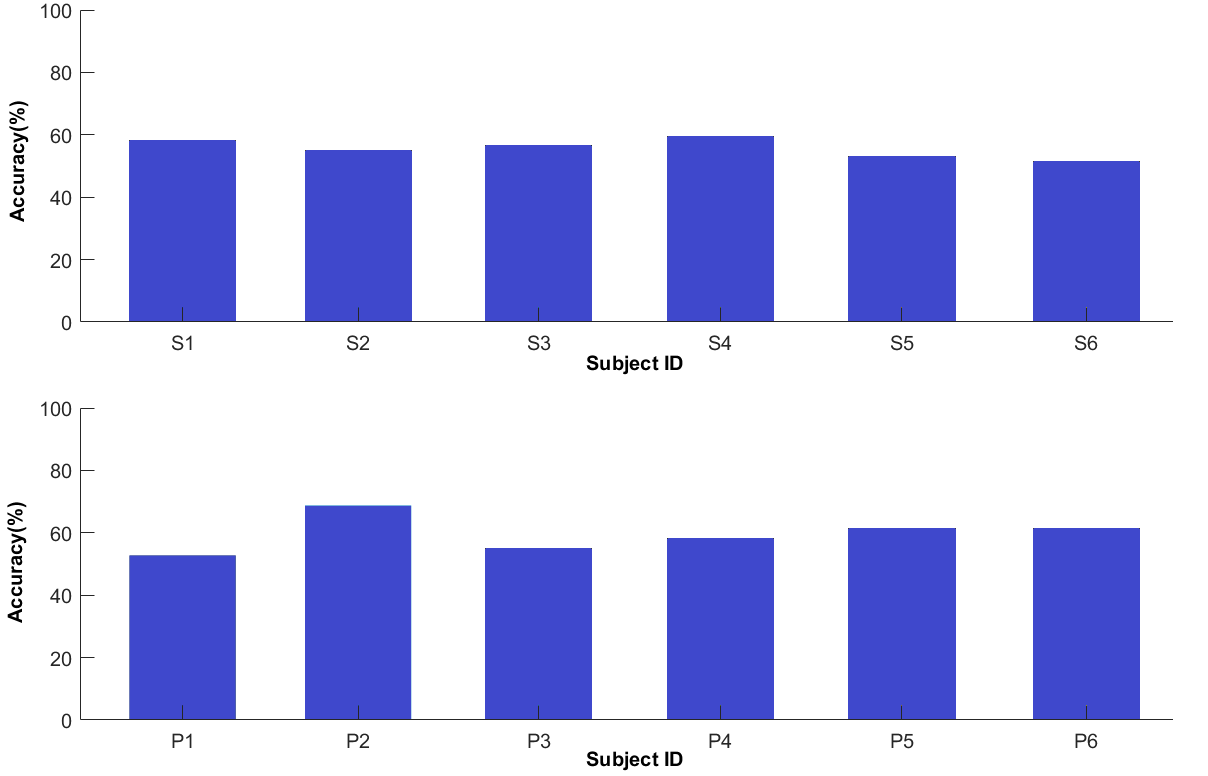

Supplement: Supplementary file 1 — Figure S1. Topographical representation of the statistically significant functional couplings (shown in Fig. 4). In the emerging graphs, the edge-width reflects the strength of the coupling and the node-size the number of edges incident to that node. The shown results correspond to Group-level analysis and reflect higher connectivity in the NMD patients. Figure S2. The classification performance in the state discrimination task (“left” vs “right”), when band-specific power-spectral density estimates are employed. Figure S3. The classification performance in the state discrimination task (“left” vs “right”), when the Common Spatial Pattern algorithm is employed in the 8–30 Hz frequency band as described by Fabien Lotte [1]. (ZIP 819 kb) [file 12984_2018_431_MOESM1_ESM.zip › Figure_S3.tif]

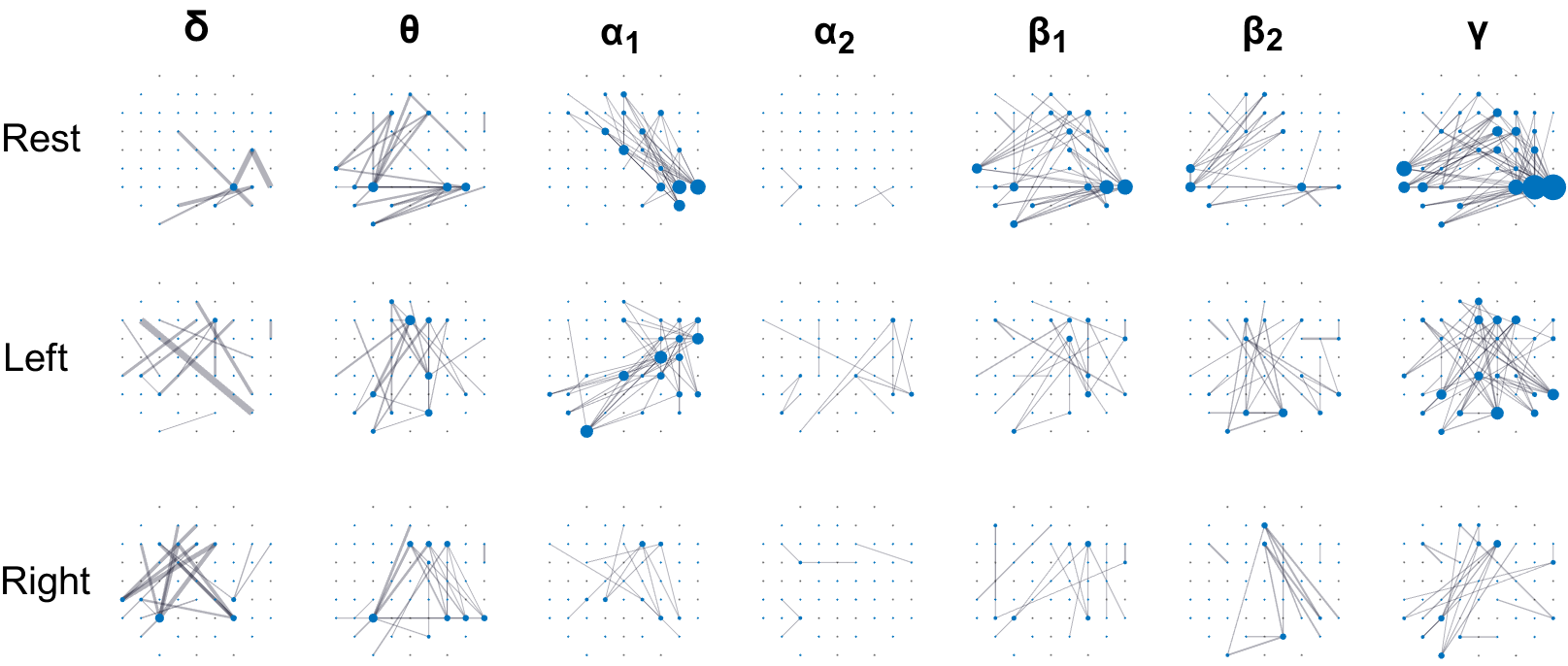

Supplement: Supplementary file 1 — Figure S1. Topographical representation of the statistically significant functional couplings (shown in Fig. 4). In the emerging graphs, the edge-width reflects the strength of the coupling and the node-size the number of edges incident to that node. The shown results correspond to Group-level analysis and reflect higher connectivity in the NMD patients. Figure S2. The classification performance in the state discrimination task (“left” vs “right”), when band-specific power-spectral density estimates are employed. Figure S3. The classification performance in the state discrimination task (“left” vs “right”), when the Common Spatial Pattern algorithm is employed in the 8–30 Hz frequency band as described by Fabien Lotte [1]. (ZIP 819 kb) [file 12984_2018_431_MOESM1_ESM.zip › Figure_S1.tif]
